# Demographic determinants of syphilis seroprevalence among U.S. blood donors, 2011–2012

**DOI:** 10.1186/s12879-015-0805-3

**Published:** 2015-02-15

**Authors:** Mark Andrew Kane, Evan Martin Bloch, Roberta Bruhn, Zhanna Kaidarova, Edward Laurence Murphy

**Affiliations:** School of Public Health, University of California Berkeley, Berkeley, CA USA; Blood Systems Research Institute, 270 Masonic Avenue, San Francisco, CA 94118 USA; Department of Laboratory Medicine, University of California San Francisco, 270 Masonic Avenue, San Francisco, CA 94118 USA; Department of Epidemiology/Biostatistics, University of California San Francisco, 270 Masonic Avenue, San Francisco, CA 94118 USA

**Keywords:** *Syphilis*, Seroprevalence, Blood donors, Public health surveillance

## Abstract

**Background:**

No cases of transfusion-transmitted syphilis have been described for over four decades. While there is mandatory transfusion screening for syphilis, the absence of transmission is in part ascribed to a low prevalence of syphilis in the blood donor population, the concomitant use of antibiotics in a high proportion of transfusion recipients, allied with poor survival of T. *pallidum* during refrigerated storage of blood products.

**Methods:**

A cross-sectional retrospective data analysis was conducted to ascertain the prevalence of *Treponema pallidum* antibodies in U.S. blood donors by demography and geography. Routine blood donation testing data and demographics were extracted from the data warehouse of a large network of U.S. blood centers. Crude and adjusted prevalence of *T. pallidum* antibodies and active syphilis infection were calculated, and GIS mapping was used to illustrate geographic distribution.

**Results:**

The prevalence of *T. pallidum* seropositivity and active syphilis in first time donors was 162.6 (95% CI 145.5-181.2) per 100,000 donors and 15.8 (95% CI 10.8-22.3) per 100,000 donors, respectively. The odds of *T. pallidum* seropositivity were significantly elevated in African American (OR = 18.9, 95% CI 14.2-25.2), and Hispanic (OR = 2.8, 95% CI 2.0-3.8) as compared to Caucasian donors. Similarly, the odds of active *T. pallidum* infections were significantly higher in African American (OR 15.0, 95% CI 7.0-32.3) and Hispanic (OR = 5.8, 95% CI 2.9-11.6) as compared to Caucasian donors. Syphilis seropositivity was associated with first time blood donation, increasing age, lower education, birth outside the US, and positive tests for HIV and HCV. Geographically, *T. pallidum* seropositivity was increased in southern and western regions of the US.

**Conclusions:**

Given the low seroprevalence of syphilis in blood donors, continued screening remains debatable; however it may provide a public health benefit through surveillance of at-risk populations.

## Background

*Treponema pallidum* subsp. *pallidum (T. pallidum)*, the causative agent of syphilis, is mainly transmitted through unprotected sexual contact and transplacental spread during pregnancy [1]. Syphilis was the first transfusion-transmitted infection to be screened following implementation of serological screening by blood banks in 1938 [2]. Prior to testing, over 100 cases of transfusion transmitted syphilis had been reported. Mandatory transfusion screening remains, yet more than 45 years have passed without a recorded case of transfusion-transmitted syphilis in the US [3-5]. Low prevalence of syphilis among blood donors, the frequent use of antibiotics in transfusion recipients, and inability of *T. pallidum* to survive in refrigerated blood products have been cited as reasons for the absence of cases [6]. The lack of transfusion-transmitted syphilis has raised questions over the merits of continued mandatory blood screening for T. *pallidum*.

Independent of transfusion risk, syphilis remains an important disease in the United States (US) where in 2011 alone, an estimated 55,400 individuals were diagnosed with primary and secondary syphilis [7]*.* Furthermore, the need to control syphilis has increased as evidence suggests that active syphilis infections potentiate transmission of HIV [8,9]. Although incidence has decreased, syphilis remains an endemic disease in high resource settings with sustained outbreaks in selected populations [10]. Specifically, surveillance data indicate that African-Americans, Hispanics, and other minority racial/ethnic groups as well as men who have sex with men (MSM) are disproportionately affected by syphilis [11]. Consequently, elimination efforts have been initiated to reduce syphilis using targeted interventions in these high risk subgroups [12,13].

Population-based syphilis seroprevalence data are important to guide mitigation efforts. The National Health and Nutrition Examination Survey (NHANES), considered the gold standard for population-based studies, is both costly and lacks continuous surveillance [14]. In contrast, blood donation offers potential for real-time surveillance and identification of high-risk groups. While blood donors overall have lower risk due to selection, the identification of “higher” risk groups within blood donors could indicate that the same groups are at higher risk within the general population. Therefore, using an existing donor-donation database, we sought to evaluate the epidemiology of syphilis in the blood donor population so as to inform both the value of continued transfusion screening as well as the benefit to public health.

## Methods

### Study population

We conducted a cross-sectional study in which syphilis test results were reviewed in combination with demographic information on allogeneic whole blood and apheresis donors that donated at all Blood Systems Inc. (BSI) collection centers from January 2011 to December 2012; autologous donors were excluded from the study. This time period was selected as it offered contemporary and complete data at time of analysis. Demographic data, which are routinely collected on donors, include self-identified race/ethnicity, age, gender, maximum educational attainment and country of birth. Both demographic information and laboratory (serologic and molecular testing) results from each blood donation were stored in a centralized data warehouse. For the purposes of the study, donors with multiple donations were assigned serological status based on their final donation during the study period. Only data from individuals who donated successfully (i.e. a unit of blood was collected) were evaluated. Donor status was categorized as first time if no previous donations were documented at BSI prior to 2011 and the donor did not return for a second donation during the study period. Repeat donor status was defined as having donated prior to or more than once during the study period. We acknowledge the possibility that donors may have previously donated at a non-BSI blood center.

Blood Systems’ donations centers supply blood products to hospitals in 18 states, which are primarily distributed in the western, northern, and southern US. Donors were geographically grouped according to their zip code of residence on the pre-donation questionnaire. For this study, the western region included all donors with residence located within CA, AZ, NV, NM, and UT. The northern region included WA, OR, ID, WY, MT, CO, KS, NE, ND, SD, MN, IA, and MO. The southern region included TX, OK, AR, LA, MS, AL, GA, FL, SC, NC, and TN.

The use of existing data without personal identifiers was considered exempt from human subjects approval by the University of California San Francisco Committee on Human Research.

### Laboratory testing

Serological testing for syphilis was performed on all blood donations using a microhemagglutination assay (MHA-TP) (Beckman Coulter PK TP System, Fujirebio Diagnostics, Inc. Brea, CA) for IgG or IgM antibodies specific for *T. pallidum*. If the index sample tested reactive, it was repeated in duplicate using the same MHA-TP assay that was employed for the initial screening. If either of the repeat samples tested reactive, the sample was labeled as repeat reactive thereby prompting quarantine of the blood product and confirmatory testing. Confirmatory testing was performed using an enzyme immunoassay (EIA) (CAPTIA™ Syphilis (T. pallidum) – G, Trinity Biotech USA, Jamestown, NY). A confirmed positive test reflects *T. pallidum* seropositivity, which includes both historical and active syphilis (treponemal screening lacks the ability to differentiate between historical vs. active syphilis infections). The samples that were confirmed as *T. pallidum* MHA seropositive were further tested using a quantitative rapid plasma reagin (qRPR) (ASI RPR Card Test for Syphilis, Manufacturer: Arlington Scientific, Inc., Springville, UT) and subsequently titered. For the purpose of this analysis, active syphilis infections were identified by samples reporting a reactive qRPR titer ≥ 1:8 dilution, consistent with previous studies of syphilis serology [13].

### Statistical analysis

Overall and subgroup-specific prevalences of *T. pallidum* seropositivity and active syphilis infection were calculated using case definitions described above. Syphilis seropositivity (positive MHA-TP and EIA) prevalence was stratified by donation history (first time vs. repeat donor) and further separated by subgroup. Demographic variables included sex, country of birth (US/non-US), race/ethnicity, age group, and maximum educational attainment. In addition, crude subgroup specific seroprevalence was calculated for active syphilis infections that were identified through blood donor testing. Because positive syphilis serology is rare (<1%), the prevalence per 100,000 individuals was calculated for each subgroup with 95% confidence intervals using the Clopper-Pearson method. Prevalence was age adjusted using the direct method to the population represented in 2001–2004 NHANES data and limited to ages under 50 years [14]. Pearson’s *χ*^2^ test was used to evaluate differences between proportions and *χ*^2^ for trend was also calculated.

In order to visualize the geographic distribution of *T. pallidum* seropositivity, ArcGIS 9.3 (Esri, Redlands, CA) was utilized. Donors were assigned states according to self-reported zip code of residence at time of donation. Only states with greater than 5,000 donors were included. States were categorized into deciles according to state seroprevalence per 100,000 donors.

Univariate logistic regression was used to compute odds ratios (ORs) for factors associated with both *T. pallidum* seropositivity and active syphilis infection. Next, multivariate logistic regression was used to produce adjusted estimates of association. Variables associated with syphilis (p < 0.10) in univariate analyses were included in the initial multivariate models, and the final multivariate models included all variables that remained significant in the model (p < 0.05). The data were analyzed using Stata/MP version 12.1 (StataCorp LP, College Station, TX).

## Results

Between January 2011 and December 2012, Blood Systems collected 1,826,745 allogeneic blood donations from 798,761 donors. On average, there were 2.3 donations per donor with a range of one to 54 donations. Donors were between 16–99 years of age with the most frequent age at donation being 18 (Table 1). There was a slight male majority. Greater than 90% of the donors reported living in the western, southern, and northern portions of the US. The majority of donors (64%) reported education beyond a high school diploma. Self-reported race/ethnicity included 62% Caucasian, 4% African American, 19% Hispanic, 3% Asian, 1% Native American, 3% other, and 9% who declined to answer.

**Table 1:** **Characteristics of the study population**

**Variable**	**Number of donors (n = 798,761)**	**(%)**
**Sex**		
Male	404,956	(51)
Female	393,798	(49)
**Age**		
16-19	161,244	(20)
20-29	164,829	(21)
30-39	114,473	(14)
40-49	118,913	(15)
50+	239,302	(30)
**Race/Ethnicity**		
Caucasian	494,913	(62)
African American	29,624	(4)
Hispanic	148,201	(19)
Asian	21,410	(3)
Native American	9,756	(1)
Other	22,091	(3)
Unknown	72,766	(9)
**Education**		
< High school	148,147	(19)
High school	107,236	(13)
Some college	267,519	(33)
Bachelors	129,969	(16)
Masters/PhD	59,859	(7)
Missing	86,031	(11)
**Country of birth**		
USA	662,390	(83)
Non-USA	54,286	(7)
Missing	82,085	(10)
**Donation history**		
First time	202,323	(25)
Repeat	596,438	(75)
**Region of residence**		
Northern	293,493	(39)
Western	239,619	(32)
Southern	214,779	(29)
**Syphilis status**		
Seropositive	436	(0.05)
Active	55	(0.007)

### Syphilis seropositivity

A total of 436 of 798,761 blood donors tested positive for *T. pallidum* seropositivity yielding an overall seroprevalence of 54.6 per 100,000 (95% CI, 49.6 to 60.0). Among first time donors, the seroprevalence of *T. pallidum* seropositivity was 162.6 per 100,000 (95% CI, 145.5-181.2) compared to 17.9 per 100,000 (95% CI, 14.7-21.7) in repeat donors. Age adjustment to the NHANES population, limited to ages under 50 years, demonstrated a prevalence of 63.1 per 100,000 overall, 217.3 per 100,000 in first time blood donors and 22.2 per 100,000 in repeat donors. A total of 55 donors were identified as having active syphilis infection as defined by a qRPR titer ≥ 1:8 yielding a seroprevalence of 6.9 per 100,000 (95% CI, 5.2 to 9.0). Table 2 examines seroprevalence of *T. pallidum* seropositivity and active infection for different subgroups of donors.

**Table 2:** **Prevalence of** ***T. pallidum*** **antibody and active syphilis infection by demographics and donation history**

	***T. pallidum*** **seropositivity — confirmed reactive treponemal tests**					**Active infections — ≥ 1.8 dilution qRPR titer**	
	**First time donors**		**Repeat donors**		**First time vs. repeat donors**	**All donors**	
**Category**	**Prevalence per 100,000 (95% CI)**	**OR (95% CI)**	**Prevalence per 100,000 (95% CI)**	**OR (95% CI)**	**OR (95% CI)**	**Prevalence per 100,000 (95% CI)**	**OR (95% CI)**
**Overall**	162.6 (145.5-181.2)	-	17.9 (14.7-21.7)	-	9.1 (7.3-11.3)	6.9 (5.2-9.0)	-
**Sex**							
Male	162.7 (138.1-190.4)	1.0	20.1 (15.4-25.9)	1.0	10.4 (7.5-14.4)	9.9 (7.1-13.5)	1.0
Female	162.6 (139.3-188.6)	1.0 (0.8-1.2)	15.8 (11.6-20.9)	1.3 (0.9-1.9)	8.1 (6.0-10.9)	3.8 (2.1-6.3)	2.1 (1.5-3.1)
**Birthplace**							
USA	152.1 (134.0-172.0)	1.0	15.4 (12.1-19.2)	1.0	9.9 (7.7-12.8)	6.3 (4.6-8.6)	1.0
Non-USA	360.1 (276.2-461.4)	2.4 (1.8-3.1)	45.9 (26.7-73.4)	3.0 (1.8-5.1)	7.9 (4.6-13.5)	11.1 (4.1-24.1)	1.7 (0.7-4.1)
Missing	68.7 (35.5-119.9)	0.5 (0.3-0.8)	21.8 (11.9-36.4)	1.4 (0.8-2.5)	3.2 (1.5-6.9)	8.5 (3.4-17.6)	1.3 (0.6-3.0)
**Race/Ethnicity**							
Caucasian	63.0 (48.7-80.3)	1.0	6.6 (4.3-9.7)	1.0	9.5 (6.0-15.0)	2.4 (1.3-4.2)	1.0
African American	1183.7 (994.7-1397.8)	19.0 (14.1-25.6)	199.5 (139.7-276.0)	30.1 (18.2-49.9)	6.0 (4.2-8.7)	57.4 (33.4-91.9)	23.7 (11.3-49.6)
Hispanic	188.1 (150.9-231.6)	3.0 (2.2-4.1)	35.5 (24.9-49.2)	5.4 (3.2-8.9)	5.3 (3.6-7.8)	16.9 (10.9-24.9)	7.0 (3.5-13.8)
Asian	75.2 (24.4-175.5)	1.2 (0.5-3.0)	6.8 (0.2-37.7)	1.0 (0.1-7.5)	11.1 (1.3-95.2)	0 (0.0-17.2)	0 (−)
Native American	118.5 (32.3-303.2)	1.9 (0.7-5.2)	15.7 (0.4-87.3)	2.4 (0.3-17.4)	7.6 (0.8-67.8)	0 (0.0-37.8)	0 (−)
Other	113.3 (48.9-223.1)	1.8 (0.9-3.7)	6.7 (0.2-37.1)	1.0 (0.1-7.4)	17.0 (2.1-136.3)	4.5 (0.1-25.2)	1.9 (0.2-14.4)
Unknown	92.6 (58.0-140.1)	1.5 (0.9-2.4)	12.2 (4.5-26.7)	1.8 (0.8-4.5)	7.6 (3.1-18.7)	0 (0.0-5.1)	0 (−)
**Age**							
16-19	16.3 (8.67-27.9)	1.0	8.6 (3.5-17.7)	1.0	1.9 (0.8-4.8)	7.4 (3.9-13.0)	1.0
20-29	116.5 (88.2-150.9)	7.2 (3.9-13.1)	29.3 (20.3-41.0)	3.4 (1.5-7.7)	4.0 (2.6-6.1)	16.4 (10.8-23.8)	2.2 (1.1-4.3)
30-39	225.8 (172.7-289.9)	13.9 (7.6-25.3)	19.4 (11.3-31.2)	2.3 (0.9-5.5)	11.6 (6.8 (19.9)	6.1 (2.5-12.6)	0.8 (0.3-2.1)
40-49	497.1 (405.9-602.5)	30.7 (17.2-54.7)	27.5 (18.1-40.0)	3.2 (1.4-7.3)	18.2 (11.9-27.8)	5.9 (2.4-12.1)	0.8 (0.3-2.0)
50+	368.1 (297.9-449.8)	22.7 (12.7-40.5)	10.3 (6.5-15.6)	1.2 (0.5-2.8)	35.9 (22.6-57.1)	0.8 (0.1-3.0)	0.1 (0.0-1.2)
		*x* ^2^ trend p < 0.0001		*x* ^2^ trend p = 0.1635			*x* ^2^ trend p < 0.0001
**Education**							
< High school	79.7 (59.3-104.7)	1.0	19.0 (10.9-30.9)	1.0	4.2 (2.4-7.4)	10.8 (6.2-17.5)	1.0
High school	427.4 (347.8-519.5)	5.4 (3.8-7.5)	20.3 (11.8-32.5)	1.1 (0.5-2.1)	21.2 (12.7-35.4)	9.3 (4.5-17.2)	0.9 (0.4-1.9)
Some college	184.7 (151.3-223.4)	2,3 (1,7-3.2)	24.3 (18.1-31.9)	1.3 (0.7-2.2)	7.6 (5.5-10.7)	8.6 (5.5-12.9)	0.8 (0.4-1.5)
BS/BA	159.4 (110.4-222.7)	2.0 (1.3-3.1)	9.2 (4.4-16.9)	0.5 (0.2-1.1)	17.4 (8.6-35.1)	3.1 (0.8-7.9)	0.3 (0.1-0.8)
MS/PhD	100.6 (71.3-152.8)	1.3 (0.8-2.1)	7.9 (2.1-20.1)	0.4 (0.1-1.2)	12.8 (3.9-41.7)	1.7 (0.04-9.3)	0.2 (0.0-1.2)
Missing	106.4 (71.3-152.8)	1.3 (0.8-2.1)	15.3 (7.0-29.1)	0.8 (0.4-1.8)	7.0 (3.3-14.7)	1.2 (0.03-6.5)	0.1 (0.0-0.8)
		*x* ^2^ trend p = 0.6597		*x* ^2^ trend p = 0.0581			*x* ^2^ trend p = 0.0003
**Donor history**							
First time						15.8 (10.8-22.3)	1.0
Repeat						3.9 (2.4-5.8)	4.1 (2.4-7.0)

Overall, unadjusted *T. pallidum* seropositivity was increased more than 9-fold in first time donors as compared to repeat donors. In addition, with the exception of the 16–19 year old age group, *T. pallidum* seropositivity was significantly (p < 0.05) greater in first time compared to repeat donors across all demographic subgroups (Table 2). Among first time donors, both African Americans (1183.7; 95% CI 994.7-1397.8) and Hispanics (188.1; 95% CI 150.9-231.6) had significantly (p < 0.0001) higher seroprevalence than Caucasians (63.0; 95% CI 48.7-80.3). The *χ*2 test for trend indicated that seroprevalence among first time donors increased with age category (p < 0.0001).

**Figure 1:**
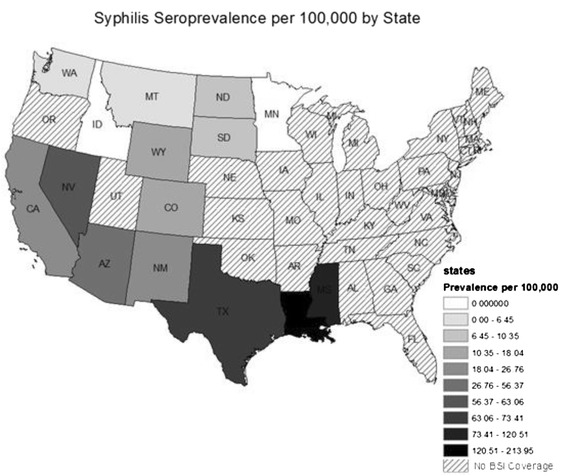
**Prevalence of** ***T. pallidum*** **seropositivity according to state of residence.** Seroprevalence (in deciles) is represented in grey-scale, with white being lowest and black highest.

Figure 1 shows the geographic distribution of *T. pallidum* seropositivity among blood donors by residence. Seroprevalence was highest in the southern states of Louisiana, Mississippi, and Texas. The majority of states located in the northern region were categorized in the lowest deciles including Oregon, Idaho, Montana, and Minnesota.

Multivariate logistic regression was performed to estimate independent associations of the various demographic and geographic factors of *T. pallidum* seropositivity (Table 3). Seropositivity was associated with first time donor status as well as African American, Hispanic, Other and Unknown race/ethnicity. African Americans had 18 times the odds of testing seropositive for *T. pallidum* as compared to Caucasians (OR 18.9; 95% CI 14.2-25.2), after adjusting for other factors. A positive HIV or HCV test result was associated with syphilis seropositivity although the confidence intervals are wide (OR 16.6; 95% CI 4.5-60.5 and OR 7.6; 95% CI 3.9-14.7, respectively). As age increased, the odds of seropositivity also increased and as education level increased, seropositivity decreased. Blood donors born within the US were less likely to have evidence for seropositivity and those residing in western and southern states were more likely to be seropositive for syphilis than those in northern states.

**Table 3:** **Independent predictors of** ***T. pallidum*** **seropositivity: odds ratios and 95% confidence intervals from the multivariate logistic regression model**

**Variable**	**OR [95% CI]**
**Age**	
16-19	1.00
20-29	11.0 [6.1-19.7]
30-39	16.7 [9.2-30.4]
40-49	41.4 [23.5-73.1]
50+	31.2 [17.6-55.3]
**Donor history**	
Repeat	1.00
First time	11.1 [8.6-14.3]
**Country of birth**	
USA	1.00
Non-USA	2.0 [1.5-2.7]
**Education**	
< High school	1.00
High school/Some college	0.6 [0.5-0.9]
Bachelors	0.4 [0.3-0.6]
Masters/PhD	0.3 [0.1-0.5]
**Race/Ethnicity**	
Caucasian	1.00
African American	18.9 [14.2-25.2]
Hispanic	2.8 [2.0-3.8]
Other	1.9 [1.1-3.3]
Missing	3.3 [1.4-7.6]
**HIV Testing**	
Negative	1.00
Positive	16.6 [4.5-60.5]
**HCV Testing**	
Negative	1.00
Positive	7.6 [3.9-14.7]
**US Region**	
Northern	1.00
Western	2.1 [1.4-3.1]
Southern	2.6 [1.8-3.8]

### Active syphilis

The prevalence of active syphilis infection (qRPR titers ≥ 1:8) was also analyzed by demographic subgroup (Table 2). Prevalence was significantly higher in males as compared to females (p = 0.001) and in first time donors as compared to repeat donors (p < 0.001). The prevalence of active syphilis infection in African Americans (57.4; 95% CI 33.4-91.9) was significantly (p < 0.0001) greater than in all other race/ethnicity subgroups; Hispanic (16.9; 95% CI 10.9-24.9) blood donors had a significantly (p < 0.0001) higher prevalence of active infection compared to Caucasians (2.4; 95% CI 1.3-4.2). Unlike the seropositivity results, the 20–29 age group had the highest prevalence of active infection while the 50+ age group had the lowest. There was also an inverse relationship between age group and active syphilis infection (p < 0.0001, *X*^2^ trend). Those without a high school diploma had significantly higher prevalence when compared to those who completed higher education (ie. BA/BS, MS/PhD, p = 0.0124, data not shown). There was an inverse relationship between educational achievement and active syphilis infections (p = 0.0003, *X*^2^ test for trend).

In a multivariate model (Table 4), active syphilis infection was independently associated with age (20–29 years), first time donor status and African American and Hispanic race/ethnicity. Females were less likely to be positive for active syphilis infection than males. A concurrent positive HIV test result was associated with increased odds of active syphilis infection although the confidence interval was wide (OR 208.0; 95% CI 56.6-764.2) (estimates were based on only three co-infections).

**Table 4:** **Independent predictors of active syphilis (qRPR > = 1:8): odds ratios and 95% confidence intervals from the multivariate logistic regression model**

**Variable**	**OR [95% CI]**
**Sex**	
Male	1.00
Female	0.4 [0.2-0.8]
**Age**	
16-19, 30+	1.00
20-29	3.1 [1.8-5.2]
**Donor history**	
Repeat	1.00
First Time	2.9 [1.7-5.1]
**Race/Ethnicity**	
Caucasian	1.00
African American	15.0 [7.0-32.3]
Hispanic	5.8 [2.9-11.6]
Other	0.6 [0.1-4.6]
**HIV Testing result**	
Negative	1.00
Positive	208.0 [56.6-764.2]

## Discussion

We report a low seroprevalence of *T. pallidum* seropositivity in US blood donors: 162.6 and 17.9 per 100,000 in first time and repeat donors, respectively. In contrast, the NHANES 2001–2004 reported an overall seroprevalence of 710 per 100,000 individuals in the US general population using a comparable testing strategy [14]. After age-adjustment, the NHANES results were still over three times higher than our first time donor population. However, lower prevalence estimates than previous national surveillance studies were observed in African American and Hispanic race/ethnicities, which is similar to NHANES and syphilis case surveillance data [14-17]. Furthermore, we noted regional differences in syphilis donor seroprevalence, which was higher in those living in the southern than western or northern regions. There were far fewer cases of active syphilis infection (15.8 per 100,000 in first time donors) than cases of seropositivity. NHANES reported a prevalence of active syphilis of 80 per 100,000 in the general population, using the same definition as our study (qRPR ≥ 1:8).

In NHANES 2001–2004 study of syphilis serology, investigators reported large racial/ethnic differences between Caucasians and African-Americans. Gottlieb et al. reported an unadjusted prevalence (seropositivity) ratio of 61:1 for African-Americans compared to Caucasians [13,14]. In contrast, the prevalence ratio of African-Americans to Caucasians in our study was 18.8:1 in first time donors. First time donors are thought to offer a better estimate of infectious risk to that of the general population given that, unlike repeat donors, they have not been tested previously and are less affected by selection bias. Nevertheless, selection bias inherent to blood donation, which excludes high-risk behaviors and favors higher educational status, may account for the observed reduced race/ethnicity ratio as compared to that of Gottlieb et al. Similarly the seropositivity prevalence ratio of first time Hispanics to Caucasian blood donors was 2.98:1. Although reduced, this still supports mitigation efforts in the African American and Hispanic communities.

The geographic distribution of *T. pallidum* seropositivity in blood donors was similar to the distribution of reported primary and secondary syphilis cases in 2012 [11]. In our study, Louisiana demonstrated the highest seroprevalence of *T. pallidum*. However, our data set included uneven sampling of blood donors in each state, which introduces potential for unstable estimates. For example, per the CDC data, a high rate of increase in primary and secondary syphilis was reported in the Northeast [11] where BSI centers are under-represented. This highlights opportunity for a collaborative effort between blood banks and state or national reporting bodies to provide more stable estimates, which are not necessarily confined to high-risk groups. For example, similar surveillance could be performed for other infections that are routinely screened in the donor population such as HIV, HBV, HCV, HTLV, West Nile Virus and *T. cruzi*.

We observed interesting patterns of age specific *T. pallidum* seropositivity and active syphilis infection. The prevalence of *T. pallidum* seropositivity increased with age. This likely reflects cumulative incidence: low level of antibodies to *T. pallidum* may be produced for decades following an infection. Because treponemal tests detect antibodies specific for *T. pallidum* antigens, treponemal tests report cumulative incidence rather than current infection. Another possible explanation for the relationship between age and *T. pallidum* seropositivity includes a birth cohort effect, whereby older cohorts may have been sexually active during times when *T. pallidum* transmission was more common. It is difficult to differentiate between these explanations without additional years of data detailing the rate of syphilis infection. For active syphilis (qRPR titers ≥1:8), a different age pattern emerged, whereby the 20–29 year age group had the highest prevalence. Young age is associated with increased sexual activity, which is a well-characterized risk factor for developing a syphilis infection [18]. Importantly, the rates of primary and secondary syphilis were highest in this same age group in the CDC surveillance data from 2012 [11].

An inverse relationship was observed between educational attainment and *T. pallidum* seropositivity. Reduced access to treatment and preventive measures may account for this finding in low socioeconomic groups. There was also a significantly higher prevalence of active syphilis in males as compared to females, which is consistent with recent reports in which the ratio of males to females with syphilis (primary and secondary) increased from 1.5 in 2000 to 8.2 in 2011 [19]. This is largely ascribed to increases in cases of syphilis among MSM [11], which suggests that MSM may be represented among blood donors despite FDA-mandated permanent deferral of any donor who has had MSM since 1977. The latter is in process of revision.

Although HIV positive donors had an increased prevalence of *T. pallidum* seropositivity and active syphilis infection compared to HIV negative donors, very few co-infections were detected resulting in unstable estimates. Other studies have confirmed that reactive syphilis serological tests or a history of genital ulceration are strongly associated with HIV infection [8,9]. HCV infection was also associated with *T. pallidum* seropositivity. Recent studies by Terrault et al. have suggested that sexual transmission of HCV among heterosexual couples is rare (1 per 190,000 sexual contacts) making sexual transmission an unlikely reason for this association [20]. Other explanations for this association include evidence that HCV infection increases the likelihood of a biological false positive test for syphilis although this is limited to qRPR tests, not specific treponemal tests [21]. HCV seropositivity is also associated with male sex and lower education, and may be a surrogate marker for injection drug use and high-risk sexual behavior.

The cost utility of mandatory screening has been questioned in the US given the low prevalence of active syphilis infection in donors and the inability of *T. pallidum* to survive refrigeration. One argument for continued screening has been the notion that syphilis is a surrogate marker for transfusion transmitted HIV. However, our study showed this not to be the case whereby we observed a very low positive predictive value of only 3 of 936 (0.32%) cases of HIV co-infection. Nonetheless, despite limited utility to blood safety in the US, syphilis screening offers public health benefit through identification of a small number of active syphilis cases. More importantly, the data it provides may be useful for population surveillance.

Our analysis had several strengths. First, it informs discussion surrounding the utility of continued syphilis screening. Second, the described testing was performed in a highly regulated environment, ensuring a high quality of laboratory data. Third, the sample size was sufficiently large to enable calculation of syphilis seroprevalence by minority population and geography.

However, our study also had limitations. First, qRPR testing lacks sensitivity in late stages of syphilis; therefore we may have underestimated the prevalence of active syphilis [22]. Second, treponemal tests are unable to differentiate syphilis from other pathogenic treponemes such as *T. pallidum* subsp. *pertenue, endemicum* and *carateum*, the causative agents of yaws, bejel, and pinta respectively [22]; however, we expect misclassification due to cross-reactivity with other treponemes to be minimal due to the rarity of such infections in the US. Third, the selection bias inherent to low risk volunteer blood donors results in underestimation of syphilis seroprevalence in the general population. However, we show that subgroup-specific differences are preserved. There may be additional geographic bias due to our use of data from the BSI network, which is focused in the western, southern and northern United States. Fourth, although the likelihood of transmission of syphilis via transfusion is low for the reasons cited (e.g. intolerance of refrigeration), one cannot discount the relative contribution of screening to reduction in risk. However, despite a changing seroprevalence, the absent transmission over four decades does support the notion that screening is only one of several factors that influence risk. Fifth, although higher rates of primary and secondary syphilis have been described in the younger age groups, we elected to retain those over age 50 given that a proportion of cases do occur in this group (most notably in males) [11]. In so doing the NHANES data may not be directly comparable given a restriction to age less than 50 years. Finally, we used operational data and no sexual risk behavior information was included in our analysis.

## Conclusions

In conclusion, the prevalence of *T. pallidum* seropositivity and active syphilis infection remains low in US blood donors. However, among donors, African-American and Hispanic race/ethnicities and residence in the southern US were associated with *T. pallidum* seropositivity. While the merits of screening blood to prevent transfusion-transmitted *T. pallidum* remain debatable, donor testing continues to offer public health benefit. Ongoing infectious disease surveillance that leverages the networks of US blood collection and testing centers offers a valuable but underutilized public health resource.

## Abbreviations

MSM: Men who have sex with men
NHANES: The National Health and Nutrition Examination Survey
BSI: Blood Systems Inc.
MHA-TP: Microhemagglutination assay
EIA: Enzyme immunoassay
qRPR: Quantitative rapid plasma reagin
OR: Odds ratio
